# Gastric cancer actionable genomic alterations across diverse populations worldwide and pharmacogenomics strategies based on precision oncology

**DOI:** 10.3389/fphar.2024.1373007

**Published:** 2024-05-02

**Authors:** Gabriela Echeverría-Garcés, María José Ramos-Medina, Rodrigo Vargas, Alejandro Cabrera-Andrade, Adriana Altamirano-Colina, María Paula Freire, Juliana Montalvo-Guerrero, Sebastián Rivera-Orellana, Paulina Echeverría-Espinoza, Luis A. Quiñones, Andrés López-Cortés

**Affiliations:** ^1^ Centro de Referencia Nacional de Genómica, Secuenciación y Bioinformática, Instituto Nacional de Investigación en Salud Pública “Leopoldo Izquieta Pérez”, Quito, Ecuador; ^2^ Latin American Network for the Implementation and Validation of Clinical Pharmacogenomics Guidelines (RELIVAF-CYTED), Santiago, Chile; ^3^ German Cancer Research Center (DKFZ), Faculty of Biosciences, Heidelberg University, Heidelberg, Germany; ^4^ Department of Molecular Biology, Galileo University, Guatemala City, Guatemala; ^5^ Escuela de Enfermería, Facultad de Ciencias de La Salud, Universidad de Las Américas, Quito, Ecuador; ^6^ Grupo de Bio-Quimioinformática, Universidad de Las Américas, Quito, Ecuador; ^7^ Cancer Research Group (CRG), Faculty of Medicine, Universidad de Las Américas, Quito, Ecuador; ^8^ Facultad de Ingenierías y Ciencias Aplicadas, Universidad Internacional SEK, Quito, Ecuador; ^9^ Laboratory of Chemical Carcinogenesis and Pharmacogenetics, Department of Basic-Clinical Oncology (DOBC), Faculty of Medicine, University of Chile, Santiago, Chile; ^10^ Department of Pharmaceutical Sciences and Technology, Faculty of Chemical and Pharmaceutical Sciences, University of Chile, Santiago, Chile

**Keywords:** gastric cancer, precision oncology, ethnic groups, genomic alterations, therapeutic strategies

## Abstract

**Introduction:** Gastric cancer is one of the most prevalent types of cancer worldwide. The World Health Organization (WHO), the International Agency for Research on Cancer (IARC), and the Global Cancer Statistics (GLOBOCAN) reported an age standardized global incidence rate of 9.2 per 100,000 individuals for gastric cancer in 2022, with a mortality rate of 6.1. Despite considerable progress in precision oncology through the efforts of international consortia, understanding the genomic features and their influence on the effectiveness of anti-cancer treatments across diverse ethnic groups remains essential.

**Methods:** Our study aimed to address this need by conducting integrated *in silico* analyses to identify actionable genomic alterations in gastric cancer driver genes, assess their impact using deleteriousness scores, and determine allele frequencies across nine global populations: European Finnish, European non-Finnish, Latino, East Asian, South Asian, African, Middle Eastern, Ashkenazi Jewish, and Amish. Furthermore, our goal was to prioritize targeted therapeutic strategies based on pharmacogenomics clinical guidelines, *in silico* drug prescriptions, and clinical trial data.

**Results:** Our comprehensive analysis examined 275,634 variants within 60 gastric cancer driver genes from 730,947 exome sequences and 76,215 whole-genome sequences from unrelated individuals, identifying 13,542 annotated and predicted oncogenic variants. We prioritized the most prevalent and deleterious oncogenic variants for subsequent pharmacogenomics testing. Additionally, we discovered actionable genomic alterations in the *ARID1A, ATM, BCOR, ERBB2, ERBB3, CDKN2A, KIT, PIK3CA, PTEN, NTRK3, TP53*, and *CDKN2A* genes that could enhance the efficacy of anti-cancer therapies, as suggested by *in silico* drug prescription analyses, reviews of current pharmacogenomics clinical guidelines, and evaluations of phase III and IV clinical trials targeting gastric cancer driver proteins.

**Discussion:** These findings underline the urgency of consolidating efforts to devise effective prevention measures, invest in genomic profiling for underrepresented populations, and ensure the inclusion of ethnic minorities in future clinical trials and cancer research in developed countries.

## Introduction

Gastric cancer is a heterogeneous disease originating in the mucus-producing cells lining the stomach interior (Ajani et al., 2017). It involves a wide range of biological components, including hormonal imbalances, ethnic backgrounds, environmental factors, epigenetics, genetic mutations, alterations in protein expression, and modifications in signaling pathways (Hanahan, 2022). According to the World Health Organization (WHO), the International Agency for Research on Cancer (IARC), and the Global Cancer Statistics (GLOBOCAN), the global age-standardized incidence rate for gastric cancer in 2022 was 9.2 per 100,000 individuals, with a mortality rate of 6.1 (Sung et al., 2021).

Since the Human Genome Project began in 1990 until the completion of the human genome sequence in 2022, genomics has played a crucial role in both foundational and translational research (Green et al., 2020; Nurk et al., 2022). Advances in high-throughput technologies, particularly in large-scale DNA sequencing, have improved our understanding of the molecular mechanisms underlying gastric cancer. This progress has been instrumental in identifying cancer driver genes (Kandoth et al., 2013; Lawrence et al., 2014), germline mutations (Lu et al., 2015), cancer driver variants in both coding and non-coding regions (Sjöblom et al., 2006; Tamborero et al., 2013; Porta-Pardo et al., 2017; Rheinbay et al., 2020), druggable proteins (Rubio-Perez et al., 2015), drug resistance mechanisms (Vasan et al., 2019), pharmacogenomics (PGx) clinical guidelines (Quinones et al., 2014; López-Cortés et al., 2020c; 2017; Varela et al., 2021), and the development of artificial intelligence algorithms (López-Cortés et al., 2020a; Jumper et al., 2021).

Genetic variants are critical in driving oncogenesis in gastric cancer, leading to the activation of oncogenes or the inactivation of tumor suppressor genes through mechanisms such as gene regulation disruption, creation of fusion genes with oncogenic properties, and alterations in the genomic architecture that globally affect gene expression (Tan and Yeoh, 2015; Yao et al., 2015).

In recent years, it has become evident that patients with the same cancer type do not uniformly respond to standard treatments (Raguz and Yagüe, 2008; Mansoori et al., 2017). Precision oncology addresses this variability by providing personalized treatment options, including appropriate medications and dosages, considering individual patient needs, their ethnicity, and treatment timing (Garraway et al., 2013; Quinones et al., 2014; Pérez-Villa et al., 2023). Therefore, identifying actionable genomic alterations is a primary goal of cancer research, particularly in the driver gene landscape of gastric cancer, to devise effective therapeutic strategies and PGx clinical guidelines (ICGC/TCGA Pan-Cancer Analysis of Whole Genomes Consortium, 2020).

Tailoring drug development to individual multi-omics profiles can enhance drug efficacy and reduce adverse reactions (López-Cortés et al., 2020c; Pérez-Villa et al., 2023; Ramos-Medina et al., 2024). Despite significant advances in precision oncology through international consortium efforts like The Cancer Genome Atlas (TCGA) and Therapeutically Applicable Research to Generate Effective Treatments (TARGET) (Spratt et al., 2016; Guerrero et al., 2018), the underrepresentation of diverse ethnic backgrounds in many cancer research studies has led to a significant bias toward Caucasians in cancer genomic databases (Guerrero et al., 2018). This bias poses a considerable obstacle to the advancement of PGx and precision oncology, especially in developing regions (Salas-Hernández et al., 2023). To bridge this gap, the primary objective of our study was to conduct an integrated *in silico* analysis to identify actionable genomic alterations in gastric cancer, evaluate their impact through deleteriousness scores and allele frequencies across nine global populations, and prioritize targeted therapeutic strategies. By integrating these findings with PGx clinical guidelines (Barbarino et al., 2018), *in silico* drug prescriptions (Tamborero et al., 2018), and clinical trial data (Ochoa et al., 2021), we aim to broaden the scope of precision oncology, ensuring it benefits a more diverse population by encouraging the development of effective and personalized therapeutic interventions.

## Materials and methods

### Study type

We conducted an integrative *in silico* analysis to understand the genomic landscape of gastric cancer across diverse ethnic populations, examine current pharmacogenomics guidelines in clinical practice, analyze *in silico* drug prescriptions of therapeutic actionable genomic alterations, and evaluate the drugs involved in early-stage and late-stage clinical trials.

### Incidence and mortality of gastric cancer

The Global Cancer Observatory (https://gco.iarc.fr/) provides a complete assessment of the global burden of cancer. By using the latest version of GLOBOCAN 2022, we identified and ranked the countries worldwide with the highest estimated age-standardized incidence and mortality rates for gastric cancer (Sung et al., 2021).

### Gastric cancer driver genes

The intOGen framework (https://www.intogen.org) is a bioinformatics resource that can identify cancer genes and determine how they work in different types of tumors (Martínez-Jiménez et al., 2020). This tool uses seven methods to identify cancer driver genes based on point mutations, namely, dNdScv (Martincorena et al., 2017), CBaSE (Weghorn and Sunyaev, 2017), MutPanning (Dietlein et al., 2020), OncodriveCLUSTL (Arnedo-Pac et al., 2019), HotMAPS (Tokheim et al., 2016), smRegions (Martínez-Jiménez et al., 2020), and OncodriveFML (Mularoni et al., 2016). Therefore, we retrieved 60 gastric cancer driver genes and identified their involvement as oncogenes (Sondka et al., 2018), tumor suppressor genes (Sondka et al., 2018), kinase genes (Manning et al., 2002; Eid et al., 2017), DNA-repair genes (Wood et al., 2001; Lange et al., 2011), RNA-binding proteins (Hentze et al., 2018), cell cycle genes (Bar-Joseph et al., 2008), metastatic genes (Zheng et al., 2018), and cancer immunotherapy genes (Patel et al., 2017).

### Identification of the oncogenic variome

The process of identifying the oncogenic variome involved two main steps. First, we extracted 275,634 single-nucleotide and insertion/deletion variants from the Genome Aggregation Database (gnomAD v4.0) (https://gnomad.broadinstitute.org/), which belonged to 60 gastric cancer driver genes (Collins et al., 2020; Karczewski et al., 2020; Nurk et al., 2022). We used the complete sequence of a human genome (GRCh38/hg38) as the reference genome (Collins et al., 2020; Karczewski et al., 2020; Nurk et al., 2022). In the second step, we used two methods, OncodriveMUT and boostDM, integrated into the Cancer Genome Interpreter (CGI) platform (https://www.cancergenomeinterpreter.org) to evaluate the tumorigenic potential of the 275,634 extracted genomic variants (Tamborero et al., 2018; Muiños et al., 2021). OncodriveMUT is a rule-based approach that considers various genomic features such as regions depleted by germline variants, gene mechanism of action, gene signals of positive selection, and clusters of somatic mutations, whereas boostDM is a machine learning-based methodology that uses *in silico* saturation mutagenesis of cancer genes to assess the oncogenic potential of mutations in human tissues. Both methods allowed us to categorize driver variants into annotated (known), predicted, and passenger mutations based on the Catalog of Validated Oncogenic Mutations (Tamborero et al., 2018; Muiños et al., 2021).

### Deleteriousness score of the oncogenic variome

Combined Annotation-Dependent Depletion (CADD) version 1.7 (https://cadd.gs.washington.edu/) serves as a machine learning-based tool that scores and classifies genetic variants to support the assessment of clinical observations. As one of the pioneering methods for the genome-wide prioritization of variants across various molecular functions, CADD was developed using more than 60 genomic features. It is capable of assessing the impact of both single-nucleotide variants and insertions/deletions (Kircher et al., 2014; Schubach et al., 2024). This tool uses a method that contrasts natural selection with simulated mutations, enabling the integration of multiple annotations into a single metric. Designed for compatibility with the GRCh38/hg38 human reference genome, CADD facilitates comprehensive genetic analysis (Rentzsch et al., 2019). In this context, we used CADD to assess the deleteriousness of annotated and predicted cancer-causing gene mutations, specifically in gastric cancer driver genes. The deleteriousness of the oncogenic variome was categorized as very high (30–50), high (25–30), medium (15–25), low (10–15), and very low (0–10).

### Functional enrichment analysis

The process of enrichment analysis provides scientists with a carefully selected explanation of sets of genes or proteins obtained from large-scale experiments in omics (López-Cortés et al., 2020b; 2022; 2021). In this study, a functional enrichment analysis was conducted on genes/proteins that drive gastric cancer and carry annotated or predicted oncogenic variants. The analysis was performed using g:Profiler version e101_eg48_p14_baf17f0 (https://biit.cs.ut.ee/gprofiler/gost) (Raudvere et al., 2019) to identify significant annotations (Benjamini–Hochberg false discovery rate (FDR) *q* < 0.001) related to biological processes in Gene Ontology (GO) (The Gene Ontology Consortium, 2021), signaling pathways in the Kyoto Encyclopedia of Genes and Genomes (KEGG) (Kanehisa and Goto, 2000), signaling pathways in Reactome (Fabregat et al., 2016), pathways in WikiPathways (Slenter et al., 2018), and Human Phenotype Ontology (HPO) (Köhler et al., 2021). Finally, signaling pathways that were significant and relevant to gastric cancer were manually curated and presented using a Manhattan plot.

### Allele frequencies in human populations

gnomAD is a collection of genomic sequencing data from various projects conducted globally (Karczewski et al., 2020). The v4.0 dataset focuses on GRCh38/hg38 and includes 730,947 exome sequences and 76,215 whole-genome sequences from unrelated individuals with different ancestral backgrounds. This study evaluated the allele frequencies of the annotated and predicted gastric cancer oncogenic variome in 9 populations, namely, African (n = 37,545), Amish (n = 456), Latino (n = 30,019), Ashkenazi Jewish (n = 14,804), East Asian (n = 22,448), European Finnish (n = 32,026), European non-Finnish (n = 590,031), Middle Eastern (n = 3,031), and South Asian (n = 45,546) (Collins et al., 2020; Karczewski et al., 2020).

### Current pharmacogenomics guidelines in clinical practice

The Pharmacogenomics Knowledge Base (PharmGKB) (https://www.pharmgkb.org/) is a database focused on pharmacogenomics. It contains important information about the relationship between genes and drugs, as well as specific guidelines for applying pharmacogenomics in clinical practice (Whirl-Carrillo et al., 2012; Barbarino et al., 2018). The database collects information from various sources, including the National Comprehensive Cancer Network (NCCN), the European Society for Medical Oncology (ESMO), the Clinical Pharmacogenetics Implementation Consortium (CPIC) (Relling and Klein, 2011; Relling et al., 2020), the Canadian Pharmacogenomics Network for Drug Safety (Ross et al., 2010), and the Royal Dutch Association for the Advancement of Pharmacy (Swen et al., 2011). In this context, we retrieved clinical annotations, gene–drug pairs, and genomic variants associated with gastric cancer pharmacogenomics guidelines.

### Therapeutic actionable genomic alterations and *in silico* drug prescription

Another CGI approach is *in silico* drug prescription. This involves identifying therapeutic actionable genomic alterations for drug response in tumors and organizing them based on their clinical importance level (Muiños et al., 2021). The method relies on two databases, the Cancer Biomarker database and the Cancer Bioactivities database, to explore the connection between the oncogenic variome and drug response (Dienstmann et al., 2015; Tamborero et al., 2018). Our study aimed to analyze the druggability of gastric cancer driver proteins carrying annotated and predicted oncogenic variants. This *in silico* analysis helped us identify the most appropriate treatment strategies based on precision oncology.

### Drugs involved in early-stage and late-stage clinical trials

The Open Targets Platform, last updated in March 2024 (https://www.targetvalidation.org), provides access to and visualization of potential therapeutic targets and drugs involved in gastric cancer clinical trials (Carvalho-Silva et al., 2019; Ochoa et al., 2021). Additionally, the Drug Repurposing Hub (https://www.broadinstitute.org/drug-repurposing-hub) serves as a bioinformatics resource to identify the mechanisms of action of drugs approved by the US Food and Drug Administration (FDA) (Corsello et al., 2017). To further elucidate the involvement of drugs in clinical trials, we used a Sankey plot, enabling a clearer distinction between drugs associated with early-stage (phases I and II) and late-stage (phases III and IV) clinical trials.

## Results

### Incidence and mortality of gastric cancer

**FIGURE 1:**
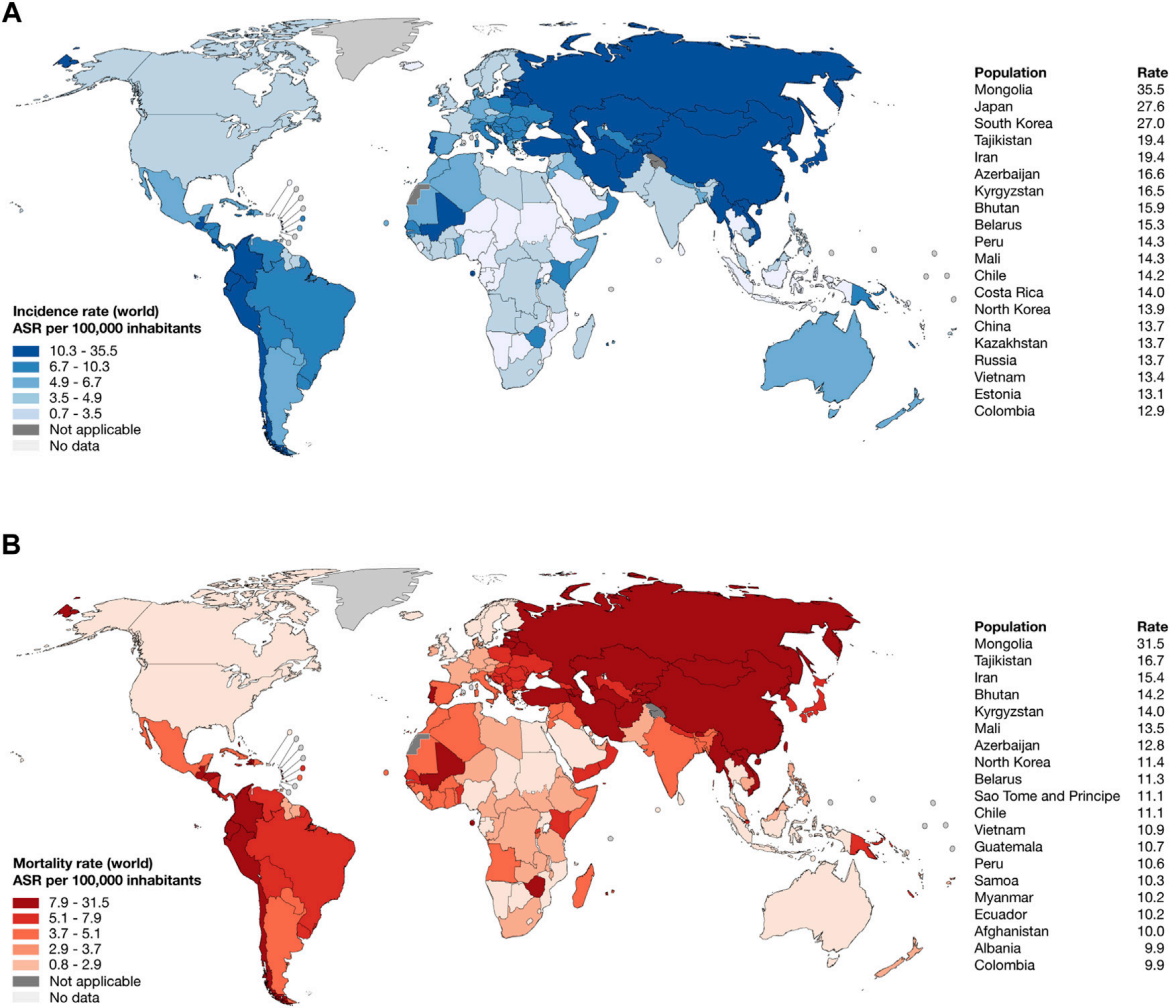
Epidemiology of stomach cancer in 2022. **(A)** Heatmap and ranking of the estimated age-standardized incidence rate of stomach cancer per 100,000 inhabitants worldwide. **(B)** Heatmap and ranking of the estimated age-standardized mortality rate of stomach cancer per 100,000 inhabitants worldwide. ASR: age-standardized rate.

According to the WHO, IARC, and GLOBOCAN 2022, the top 10 countries worldwide with the highest estimated age-standardized incidence rates of gastric cancer per 100,000 inhabitants were Mongolia (35.5), Japan (27.6), South Korea (27.0), Tajikistan (19.4), Iran (19.4), Azerbaijan (16.6), Kyrgyzstan (16.5), Bhutan (15.9), and Belarus (15.3) (Figure 1A; Supplementary Table S1); meanwhile, the top 10 countries worldwide with the highest estimated age-standardized mortality rate were Mongolia (31.5), Tajikistan (16.7), Iran (15.4), Bhutan (14.2), Kyrgyzstan (14.0), Mali (13.5), Azerbaijan (12.8), North Korea (11.4), Belarus (11.3), and Sao Tome and Principe (11.1) (Figure 1B; Supplementary Table S2) (Sung et al., 2021).

### Gastric cancer driver genes

We retrieved 60 gastric cancer driver genes from the intOGen framework (Martínez-Jiménez et al., 2020), of which 33 (55%) were tumor suppressor genes (Sondka et al., 2018), 33 (55%) were metastatic genes (Zheng et al., 2018), 15 (25%) were oncogenes (Sondka et al., 2018), 11 (18%) were kinase genes (Manning et al., 2002; Eid et al., 2017), 5 (8%) encoded RNA-binding proteins (Hentze et al., 2018), 4 (7%) were cancer immunotherapy genes (Patel et al., 2017), 2 (3%) were DNA-repair genes (Wood et al., 2001; Lange et al., 2011), and 2 (3%) were cell cycle genes (Bar-Joseph et al., 2008) (Supplementary Table S3).

### Identification of the gastric oncogenic variome and its deleteriousness scores

**FIGURE 2:**
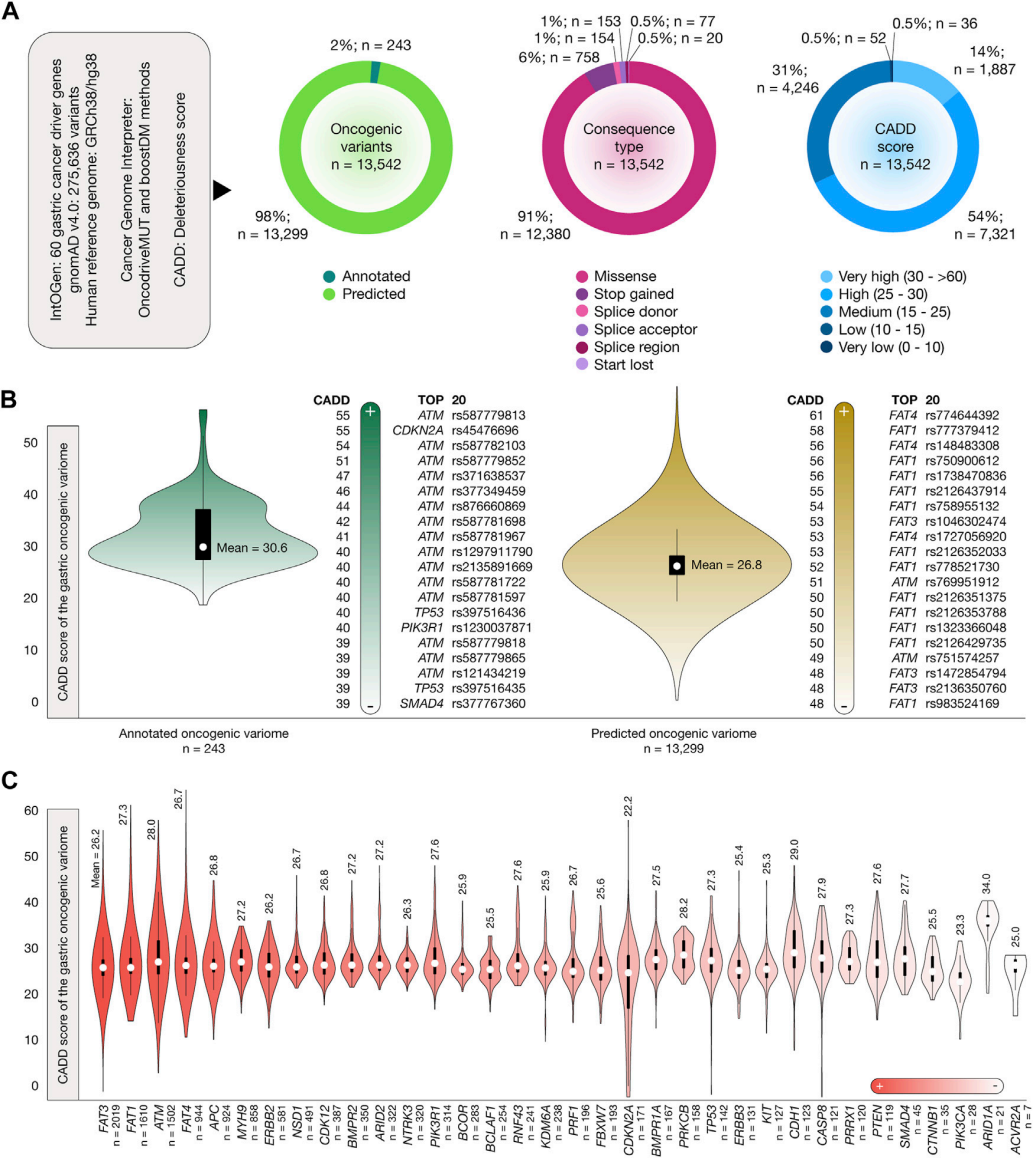
Stomach cancer driver genes, oncogenic variants, and Combined Annotation-Dependent Depletion (CADD) deleteriousness scores. **(A)** Features of stomach cancer driver genes, oncogenic variants, consequence type, and CADD deleteriousness scores. **(B)** Bean plots of CADD deleteriousness scores of the gastric oncogenic variome and ranking of annotated and predicted oncogenic variants with the highest CADD deleteriousness scores. **(C)** Ranking of the gastric cancer driver genes with the highest number of oncogenic variants and their mean CADD deleteriousness scores.

Figure 2A presents the results of the OncodriveMUT and boostDM analyses used to identify the oncogenic variome of 60 gastric cancer driver genes by using the GRCh38/hg38 human reference genome. After analyzing 275,634 variants, we identified 13,542 oncogenic variants, of which 243 (2%) were annotated and 13,299 (98%) were predicted. The consequence type analysis revealed that 12,380 (91%) were missense variants, 758 (6%) were stop-gained variants, 154 (1%) were splice-donor variants, 153 (1%) were splice-acceptor variants, 77 (0.5%) were splice region variants, and 20 (0.5%) were start-lost variants (Supplementary Table S4). Regarding the deleteriousness score, 1,887 (14%) oncogenic variants had very high CADD scores, 7,321 (54%) oncogenic variants had high CADD scores, and 4,246 (31%) had medium CADD scores. Figure 2B displays the violin plots and ranking of CADD scores of the annotated and predicted oncogenic variome related to gastric cancer driver genes. The mean CADD score of the annotated oncogenic variants was 30.6, and the annotated oncogenic variant with the highest CADD score was *ATM* rs587779813 (score = 55). The mean CADD score of the predicted oncogenic variants was 26.8, and the predicted oncogenic variant with the highest CADD score was *FAT4* rs774644392 (score = 61). The details of the ranking of the 13,542 oncogenic variants are given in Supplementary Table S5. Finally, Figure 2C provides details on the number of annotated and predicted oncogenic variants per gastric cancer driver gene. Genes with the highest number of oncogenic variants were *FAT3* (n = 2,019), *FAT1* (n = 1,610), and *ATM* (n = 1,502).

### Functional enrichment analysis

**FIGURE 3:**
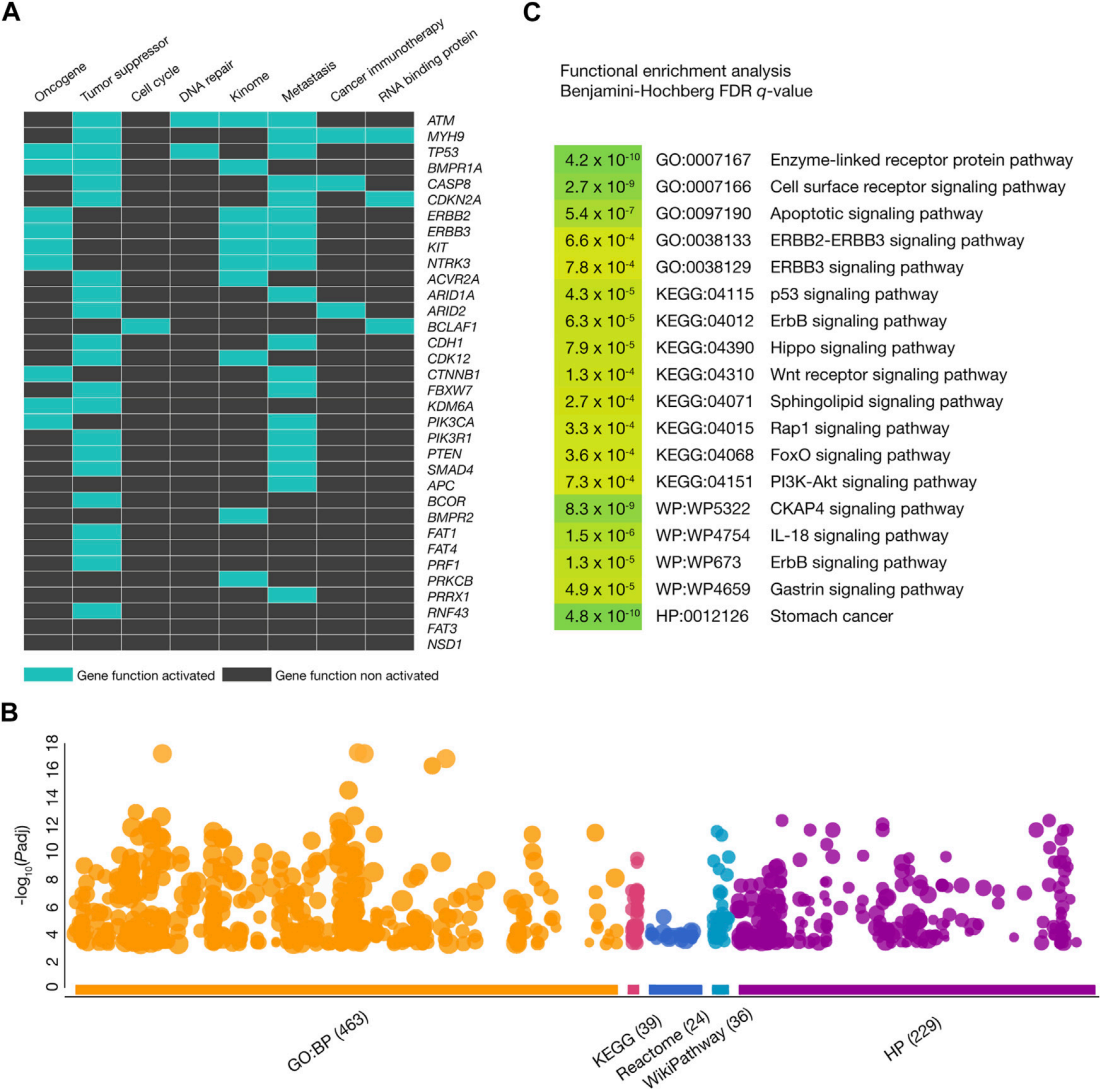
Functional enrichment analysis. **(A)** Heatmap of gastric cancer driver genes with oncogenic variants being part of oncogenes, tumor suppressor genes, cell cycle genes, DNA-repair genes, kinome, metastatic genes, cancer immunotherapy genes, and genes encoding RNA-binding proteins. **(B)** Most significant (Benjamini–Hochberg false discovery rate (FDR) *q*-value <0.001) Gene Ontology (GO) biological processes, Kyoto Encyclopedia of Genes and Genomes (KEGG) signaling pathways, WikiPathways, and Human Phenotype Ontology annotations where the gastric cancer driver genes with oncogenic variants were involved. **(C)** Manhattan plot of the most significant GO biological processes, KEGG signaling pathways, Reactome signaling pathways, WikiPathways, and Human Phenotype Ontology annotations.

Figure 3A displays a heatmap of the 34 gastric cancer driver genes carrying annotated and predicted oncogenic variants involved in several tumorigenic processes. We identified 21 tumor suppressor genes, 19 metastatic genes, 9 oncogenes, 10 kinome genes, 3 genes encoding RNA-binding proteins, 3 cancer immunotherapy genes, 2 DNA-repair genes, and 1 cell cycle gene. Figure 3B shows a Manhattan plot of the functional enrichment analysis performed on the 34 gastric cancer driver genes with annotated and predicted oncogenic variants by using g:Profiler software (Raudvere et al., 2019). We identified 463 GO biological processes (The Gene Ontology Consortium, 2021), 39 KEGG signaling pathways (Kanehisa and Goto, 2000), 24 Reactome signaling pathways (Fabregat et al., 2016), 36 WikiPathways (Slenter et al., 2018), and 229 HP ontologies (Köhler et al., 2021). Subsequently, some of the most significant (Benjamini–Hochberg FDR *q* < 0.001) annotations related to gastric cancer were cell surface receptor (GO:0007166), apoptotic (GO:0097190), p53 (KEGG:04115), ErbB (KEGG:04012), Hippo (KEGG:04390), Wnt (KEGG:04310), sphingolipid (KEGG:04071), Rap1 (KEGG:04015), FoxO (KEGG:04068), PI3K-Akt (KEGG:04151), CKAP4 (WP:WP5322), and IL-18 (WP:WP4754) signaling pathways. Lastly, the stomach cancer annotation was significant as a Human Phenotype Ontology (HPO:0012126) (Figure 3C; Supplementary Table S6).

### Deleteriousness scores and allele frequencies across human populations

**FIGURE 4:**
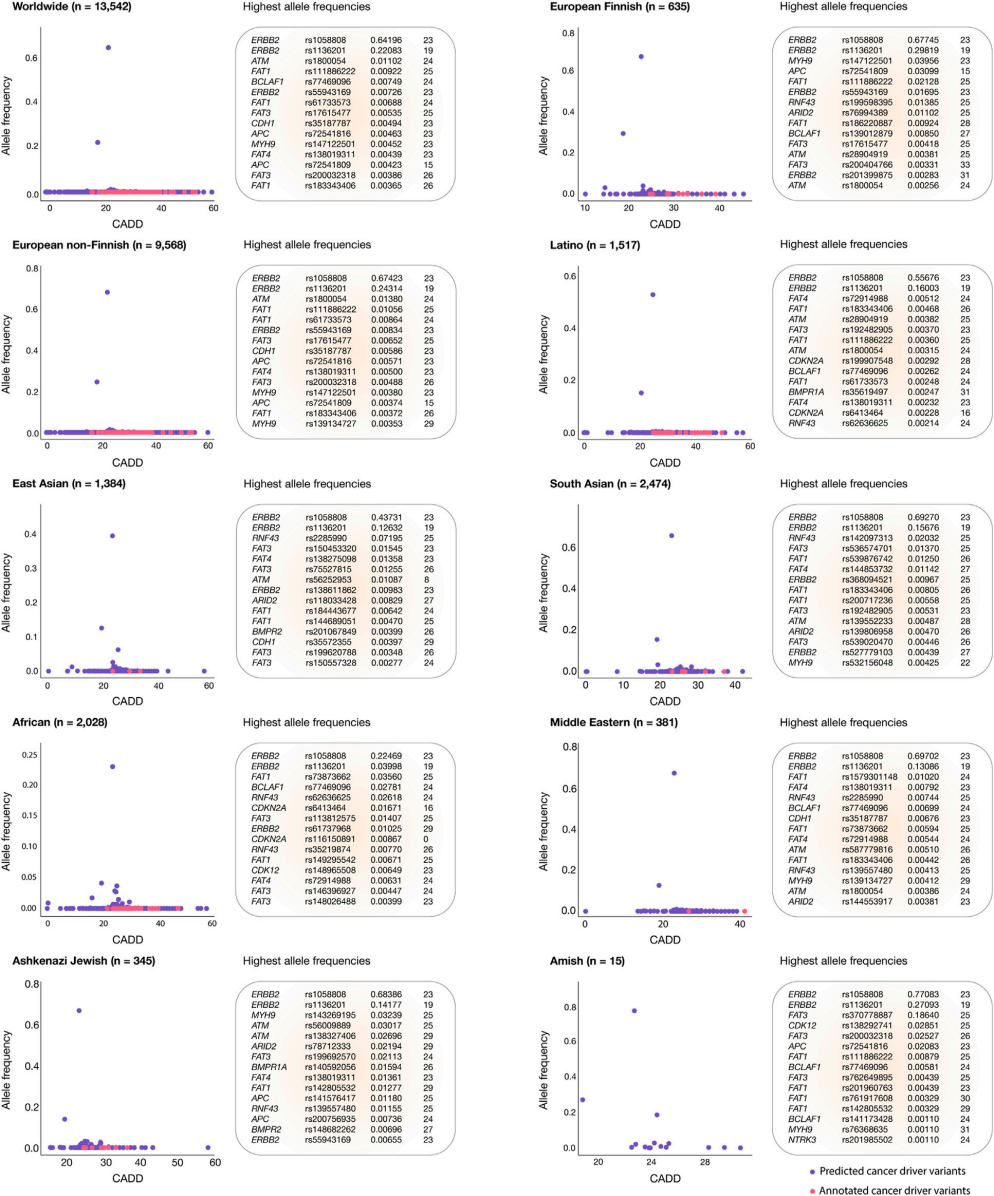
Gastric cancer oncogenic variants with the highest allele frequencies and CADD deleteriousness scores. Scatter plots and ranking of the annotated and predicted oncogenic variants with the highest allele frequencies and CADD deleteriousness scores from the European Finnish, European non-Finnish, Latino, East Asian, South Asian, African, Middle Eastern, Ashkenazi Jewish, and Amish populations.

Figure 4 shows scatter plots identifying oncogenic variants with the highest allele frequencies and the most deleterious CADD scores for each human population. The European Finnish and European non-Finnish populations had the highest mean CADD scores (26.9), followed by South Asian (26.6), Latino (26.3), East Asian (26.3), African (26.2), Middle Eastern (25.9), Amish (25.1), and Ashkenazi Jewish (25.0) populations. Worldwide, the top five oncogenic variants with the highest allele frequencies were *ERBB2* rs1058808 (0.64196), *ERBB2* rs1136201 (0.22083), *ATM* rs1800054 (0.01102), *FAT1* rs111886222 (0.00922), and *BCLAF1* rs77469096 (0.00749). The *ERBB2* rs1058808 and *ERBB2* rs1136201 oncogenic variants displayed the highest allele frequencies in the European Finnish (0.67745; 0.29819), European non-Finnish (0.67423; 0.24314), Latino (0.55676; 0.16603), East Asian (0.43731; 0.12632), South Asian (0.69270; 0.15676), African (0.22469; 0.03998), Middle Eastern (0.69702; 0.13086), Ashkenazi Jewish (0.68386; 0.14177), and Amish (0.77083; 0.27093) populations. Lastly, Figure 4 and Supplementary Table S5 provide a comprehensive ranking of oncogenic variants with the highest allele frequencies and CADD scores per human population.

### Current pharmacogenomics guidelines in clinical practice

**FIGURE 5:**
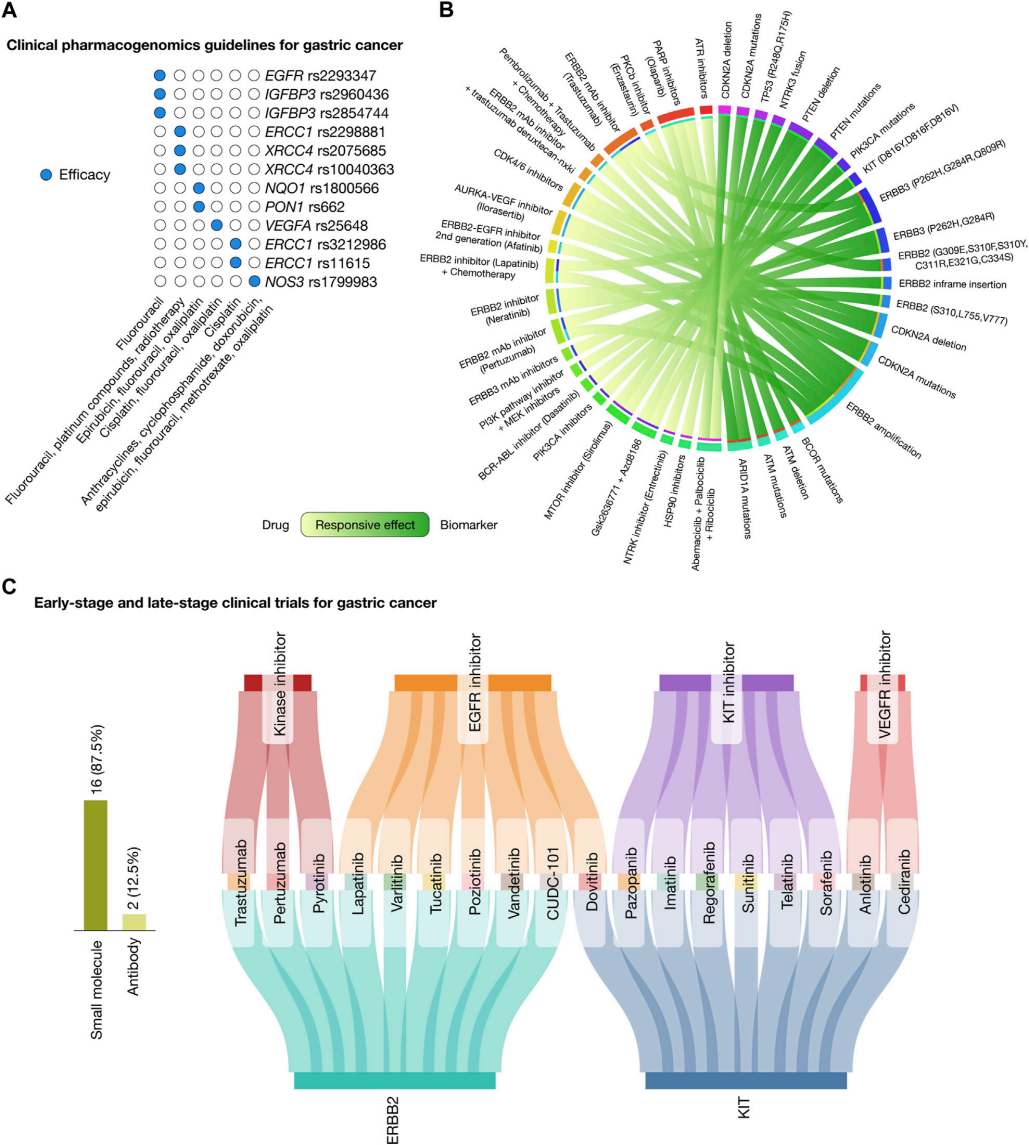
Landscape of therapeutic strategies based on precision oncology. **(A)** Current clinical pharmacogenomic guidelines for gastric cancer. **(B)** Circos plot showing *in silico* drug prescriptions of responsive effects targeting gastric cancer actionable genomic alterations. **(C)** Sankey plot of early-stage and late-stage clinical trials for gastric cancer connecting therapeutic targets, drugs, and mechanisms of action.

The PharmGKB compiles clinical guidelines, drug labels, genotype–phenotype correlations, and actionable target–drug associations (Whirl-Carrillo et al., 2012; Barbarino et al., 2018). In relation to gastric cancer, it currently lists 12 clinical annotations that involve 9 genes, 12 variants, and 6 drugs, which include combination therapies. Fluorouracil is effective in patients carrying the *EGFR* rs2293347, *IGFBP3* rs2960436, and *IGFBP3* rs2854744 variants. A combination of fluorouracil, platinum compounds, and radiotherapy is effective for patients with the *ERCC1* rs2298881, *XRCC4* rs2075685, and *XRCC4* rs10040363 variants. Epirubicin, fluorouracil, and oxaliplatin have demonstrated efficacy in patients with *NQO1* rs1800566 and *PON1* rs662 variants. Patients with the *VEGFA* rs25648 variant respond to a combination of cisplatin, fluorouracil, and oxaliplatin. Cisplatin is effective for patients with *ERCC1* rs3212986 and *ERCC1* rs11615 variants. Lastly, a regimen combining anthracyclines, cyclophosphamide, doxorubicin, epirubicin, fluorouracil, methotrexate, and oxaliplatin is effective for patients with the *NOS* rs1799983 variant (Figure 5A; Supplementary Table S7).

### *In silico* drug prescription based on precision oncology

Figure 5B presents a Circos plot identifying potential biomarkers for drug response in gastric cancer treatments according to the Cancer Genome Interpreter and the Cancer Biomarker database (Dienstmann et al., 2015; Tamborero et al., 2018). Patients with *ARID1A* oncogenic mutations have responsive effects with ATR inhibitors and olaparib; *AMT* oncogenic mutations and deletions with olaparib; *BCOR* oncogenic mutations with enzastaurin; *ERBB2* amplification with trastuzumab, afatinib, pertuzumab, a combination of lapatinib and chemotherapy, and a combination of pembrolizumab, trastuzumab, and chemotherapy; *ERBB2* mutations (G309E, S310F, S310Y, C311R, E321G, and C334S) and *ERBB2* inframe insertions (P780GSP, 781GSP, A775YVMA, and G776YVMA) with neratinib; *CDKN2A* oncogenic mutations and deletions with CDK4/6 inhibitors and ilorasertib; *ERBB3* mutations (P262H, G284R, and Q809R) with ERBB3 inhibitors, trastuzumab, pertuzumab, a combination of lapatinib and chemotherapy, and a combination of the PI3K pathway inhibitor and MEK inhibitors; *KIT* mutations (D816Y, D816F, and D816V) with dasatinib; *PIK3CA* oncogenic mutations with PIK3CA inhibitors; *PTEN* oncogenic mutations and deletions with sirolimus, and a combination of Gsk2636771 and Azd8186; *NTRK3* fusion with entrectinib; *TP53* mutations (R248Q and R175H) with HSP90 inhibitors; and lastly, *CDKN2A* oncogenic mutations and deletions with a combination of abemaciclib, palbociclib, and ribociclib (Supplementary Table S8) (Dienstmann et al., 2015; Tamborero et al., 2018).

### Drugs involved in early-stage and latestage clinical trials

The Open Targets Platform provides insights into the ongoing phase I, II, III, and IV clinical trials focusing on gastric cancer driver proteins (Carvalho-Silva et al., 2019; Ochoa et al., 2021), while the Drug Repurposing Hub details the mechanism of action of the FDA-approved drugs (Corsello et al., 2017). Figure 5C depicts a Sankey plot, representing 27 clinical trial events encompassed by 2 druggable proteins (ERBB2 and KIT), 18 drugs (comprising 84% small molecules and 16% antibodies), and 4 mechanisms of action, namely, kinase inhibitors (trastuzumab, pertuzumab, and pyrotinib), EGFR inhibitors (lapatinib, varlitinib, tucatinib, poziotinib, vandetanib, CUDC-101, and dovitinib), KIT inhibitors (pazopanib, imatinib, regorafenib, sunitinib, telatinib, and sorafenib), and VEGFR inhibitors (anlotinib and cediranib) (Supplementary Table S9).

## Discussion

In the realm of precision oncology for gastric cancer, the focus on personalized therapeutic strategies, tailored to the unique molecular profile of an individual’s tumor, represents a significant shift in cancer treatment paradigms (Liu and Meltzer, 2017). Instead of a one-size-fits-all approach, precision oncology highlights the necessity of a deep understanding of the tumor genetic landscape, recognizing that each patient may exhibit unique targets amenable to specific treatments (Le Tourneau et al., 2019). The use of bioinformatics tools is essential in interpreting the vast amount of data produced by omics technologies, thereby uncovering actionable insights that can guide treatment decisions (Valencia and Hidalgo, 2012).

The importance of precision oncology goes beyond simply identifying molecular targets; it involves integrating these insights into a comprehensive treatment strategy that takes into account the tumor microenvironment, clinical history, genetic predisposition, and lifestyle (Le Tourneau et al., 2019). This strategy facilitates a more refined approach to gastric cancer treatment, transitioning from conventional chemotherapy regimens to targeted therapies that offer greater efficacy and fewer side effects.

However, the adoption of precision oncology encounters significant obstacles, notably in developing countries. These challenges encompass the formulation of extensive PGx clinical guidelines to guide treatment decisions across a culturally and genetically varied patient base. Moreover, there is a pressing need for rigorous cost-effectiveness analysis, suitable regulatory frameworks, and increased gene/drug trials (Quinones et al., 2014; López-Cortés et al., 2017; Salas-Hernández et al., 2023).

Additionally, the lack of representation of minority populations in genomic studies poses a considerable limitation to the universal applicability of precision oncology. The majority of the genomic data available today are derived from Caucasian populations, which distorts our understanding of cancer genetics and constrains the efficacy of targeted therapies across different ethnic groups (Guerrero et al., 2018). Overcoming this discrepancy necessitates a deliberate effort to include more diverse populations in genomic research, alongside investments in genomic testing infrastructure in underrepresented regions.

Our study contributes to this domain by pinpointing actionable genomic alterations in gastric cancer, analyzing allele frequencies across populations worldwide, and optimizing therapeutic strategies in precision oncology. In this context, we analyzed 275,634 single-nucleotide and insertion/deletion variants across 60 recognized gastric cancer driver genes from 730,947 exome sequences and 76,215 whole-genome sequences from unrelated individuals, spanning a broad spectrum of ethnic backgrounds, including African, Amish, Latino, Ashkenazi Jewish, East Asian, European Finnish, European non-Finnish, Middle Eastern, and South Asian. Such a diverse dataset underscores our commitment to inclusivity in genomic research. Our findings highlighted 13,542 oncogenic variants, with a subset showing elevated deleteriousness scores. Through a functional enrichment analysis of the 34 gastric cancer driver genes carrying oncogenic variants, we found biological significance to crucial molecular mechanisms involved in the development of gastric cancer, including apoptotic, ERBB2-ERBB3, p53, Hippo, Wnt, sphingolipid, Rap1, FoxO, PI3K-Akt, CKAP4, IL-18, and gastrin signaling pathways (Lei et al., 2022).

Understanding the impact of oncogenic variants across different ethnic groups is crucial as these variants can significantly influence the susceptibility to gastric cancer (Cordova-Delgado et al., 2021; Gonzalez-Hormazabal et al., 2021; Landeros et al., 2021). This knowledge is essential for prioritizing therapeutic strategies and making informed decisions concerning healthcare economics, public health policies, and global preventive measures. To this end, we calculated the allele frequencies of gastric cancer oncogenic variants across each ethnic group, uncovering notable differences and identifying several predominant oncogenic variants worldwide. In our findings, the European non-Finnish population had 9,568 oncogenic variants with allele frequencies greater than 0; South Asians had 2,474; Africans had 2,028; Latinos had 1,517; East Asians had 1,384; European Finnish had 635; Middle Eastern populations had 381; Ashkenazi Jewish had 345; and the Amish population had 15. Notably, the most widespread oncogenic variants globally were *ERBB2* rs1058808 (0.64196) and *ERBB2* rs1136201 (0.22083). Among specific ethnic groups, the most prevalent in the European non-Finnish population was *ATM* rs1800054 (0.01380), in South Asians was *RNF43* rs142097313 (0.02032), in Africans was *FAT1* rs73873662 (0.03560), in Latinos was *FAT4* rs191329848 (0.00512), in East Asians was *RNF43* rs2285990 (0.07195), in European Finnish was *MYH9* rs147122501 (0.03956), in the Middle Eastern population was *FAT1* rs1579301148 (0.01020), in Ashkenazi Jewish was *MYH9* rs143269195 (0.03239), and in the Amish population was *FAT3* rs370778887 (0.18640). These findings underscore the importance of a comprehensive understanding of the effect of these variants to develop preventive strategies for healthy populations and craft effective therapeutic approaches for patients with gastric cancer (Pirmohamed, 2023).

A primary goal in deciphering the cancer genome is understanding the impact of oncogenic variants on the effectiveness of anti-cancer treatments. By integrating our discoveries with PGx clinical guidelines (Barbarino et al., 2018), *in silico* drug prescriptions (Tamborero et al., 2018), and data from both early-stage and late-stage clinical trials (Ochoa et al., 2021), we aim to broaden the scope of precision oncology across human populations. This initiative seeks to enhance the detection of oncogenic variants in individuals with gastric cancer and those predisposed to it, thereby facilitating the creation of more personalized treatment plans. In our thorough analysis, we identified 11 key therapeutically actionable targets carrying 3,362 annotated and predicted oncogenic variants with the highest deleteriousness scores and allele frequencies across human populations. These targets exhibited variant distributions across ethnic groups and were correlated with specific treatments. Europeans of non-Finnish descent had 2,406 oncogenic variants, South Asians had 560 variants, Africans had 492 variants, Latinos had 368 variants, East Asians had 318 variants, and Europeans of Finnish descent had 164 variants in the *ATM*, *ERBB2*, *ERBB3*, *ARID1A*, *PTEN*, *TP53*, *CDKN2A*, *NTRK3*, *PIK3CA*, *KIT*, and *BCOR* therapeutic targets. Ashkenazi Jews had 90 variants in *ATM*, *ERBB2*, *BCOR*, *PTEN*, *TP53*, *KIT*, *NTRK3*, *ERBB3*, *CDKN2A*, and *BCOR*. The Middle Eastern population had 89 variants in *ATM*, *ERBB2*, *ERBB3*, *BCOR*, *CDKN2A*, *PTEN*, and *NTRK3*. Lastly, the Amish population had three variants in the *ERBB2* and *NTRK3* therapeutic targets.

Regarding responsive treatments analyzed through *in silico* drug prescriptions, ATR and PARP inhibitors, particularly olaparib, target *ARID1A* oncogenic mutations and *AMT* oncogenic mutations and deletions. Treatments for ERBB2 amplifications include trastuzumab, afatinib, pertuzumab, a combination of lapatinib and chemotherapy, and a combination of pembrolizumab, trastuzumab, and chemotherapy. Neratinib is effective for certain *ERBB2* mutations (G309E, S310F, S310Y, C311R, E321G, and C334S) and *ERBB2* inframe insertions (P780GSP, 781GSP, A775YVMA, and G776YVMA). CDK4/6 inhibitors and ilorasterbin are used for *CDKN2A* mutations and deletions. For specific ERBB3 mutations (P262H, G284R, and Q809R), treatments include ERBB3 inhibitors, trastuzumab, pertuzumab, combinations of lapatinib and chemotherapy, and combinations of PI3K pathway inhibitors and MEK inhibitors. Dasatinib targets specific *KIT* mutations (D816Y, D816F, and D816V); PIK3CA inhibitors are used for *PIK3CA* oncogenic mutations; sirolimus and a combination of Gsk2636771 and Azd8186 target *PTEN* mutations and deletions; entrectinib targets *NTRK3* fusion; HSP90 inhibitors address certain *TP53* mutations (R248Q and R175H); and a combination of abemaciclib, palbociclib, and ribociclib targets *CDKN2A* oncogenic mutations (Dienstmann et al., 2015; Tamborero et al., 2018). Regarding clinical trials, 27 events are investigating 18 potential drugs targeting the ERBB2 and KIT therapeutic targets. Trastuzumab and pertuzumab kinase inhibitors are under evaluation in phase III clinical trials targeting ERBB2 (NCT01774786 and NCT01041404) (Van Cutsem et al., 2015; Tabernero et al., 2023). Meanwhile, other EGFR inhibitors, KIT inhibitors, and VEGFR inhibitors are being evaluated in phases I and II clinical trials. This comprehensive approach, which integrates genomic data analysis with targeted *in silico* drug prescriptions and clinical trial agents, marks a significant advancement in precision oncology. It paves the way for more effective and personalized treatment options for patients with gastric cancer and underscores the potential for preventive strategies in at-risk populations across different ethnic groups.

Despite the significant advances made in precision oncology for gastric cancer, several limitations persist. The underrepresentation of minority populations in genomic studies restricts the universal applicability of findings as most genomic data are derived from Caucasian populations (Spratt et al., 2016; Guerrero et al., 2018). This disparity distorts our understanding of cancer genomics and limits the effectiveness of targeted therapies across diverse ethnic groups. Furthermore, the implementation of precision oncology, especially in developing countries, faces obstacles such as the need for comprehensive pharmacogenomics clinical guidelines, cost-effectiveness analysis, suitable regulatory frameworks, and increased gene/drug trails. These challenges highlight the urgent need for a more inclusive approach to genomic research and the development of tailored treatment strategies that consider the unique genetic landscape of each patient with cancer.

Looking ahead, it is imperative to broaden the inclusion of diverse populations in genomic studies, alongside investments in genomic testing infrastructure in regions currently underrepresented. Future research should focus on the database of oncogenic variants across varied ethnicities to forge more effective and personalized treatment options for patients with gastric cancer. There lies a promising path in leveraging *in silico* drug prescriptions and clinical trial data to further refine treatment strategies. The incorporation of these developments with PGx clinical guidelines is crucial for the optimization of therapeutic approaches and the creation of effective preventive strategies for at-risk populations across different ethnic groups. Additionally, enhancing investments in genetics research, particularly in developing regions, and efforts to include ethnic minorities in clinical trials and cancer research will be crucial (Paz-y-Miño et al., 2010; Paz-Y-Miño et al., 2015; Paz-Y-Miño et al., 2016; López-Cortés et al., 2018).

In conclusion, our study marks a significant advancement in applying precision oncology to gastric cancer, underscoring the critical role of genetic diversity in enhancing therapeutic strategies. Through the analysis of a wide range of oncogenic variants across diverse populations, we established a foundation for treatments that are both more equitable and effective. Integrating our discoveries with PGx clinical guidelines, *in silico* drug prescriptions, and clinical data heralds a new era of personalized cancer care. Nevertheless, overcoming existing limitations and adopting future perspectives require a unified global effort to ensure that precision oncology serves patients globally (Iorio et al., 2016; Pirmohamed, 2023). As we progress toward a more inclusive and personalized approach to cancer treatment, the transformative potential of precision oncology to improve patient care and outcomes becomes increasingly evident.

## Data Availability

The original contributions presented in the study are included in the article/Supplementary Material; further inquiries can be directed to the corresponding author.
